# Effect of Thermal Treatments on Sn-Alloyed Al-Mg-Si Alloys

**DOI:** 10.3390/ma12111801

**Published:** 2019-06-03

**Authors:** Florian Schmid, Peter J. Uggowitzer, Robin Schäublin, Marion Werinos, Thomas Ebner, Stefan Pogatscher

**Affiliations:** 1 Christian Doppler Laboratory for Advanced Aluminum Alloys, Chair of Nonferrous Metallurgy, Montanuniversitaet Leoben, Franz-Josef Straße 18, 8700 Leoben, Austria; florian.schmid@unileoben.ac.at; 2 Chair of Nonferrous Metallurgy, Montanuniversitaet Leoben, Franz-Josef-Strasse 18, 8700 Leoben, Austria; peter.uggowitzer@unileoben.ac.at (P.J.U.); marion.werinos@gmail.com (M.W.); 3 Laboratory of Metal Physics and Technology, Department of Materials, ETH Zurich, 8093 Zurich, Switzerland; robin.schaeublin@scopem.ethz.ch; 4 AMAG rolling GmbH, Postfach 32, 5282 Ranshofen, Austria; thomas.ebner@amag.at

**Keywords:** aluminum alloys, aging, vacancies, microalloying

## Abstract

Sn-alloying, by deploying comparatively high vacancy binding energy, mitigates the undesired natural aging behavior of 6xxx-alloys. Targeted selection of pre-aging parameters can have a positive influence on natural aging and paint-bake performance. In this study, we aimed to combine the two approaches of Sn-alloying and pre-aging. Our results indicate that alloys modified with 100 at.-ppm Sn require altered heat treatment. In terms of solution aging and quenching, we show that the cooling rate needed depends on the types of alloy. The rate must be adapted, according to the number of intermetallic particles, to guarantee a sufficiently high level of Sn atoms in solid solution. The rather high number of intermetallic phases in alloy EN-AW-6061 means that it requires fast quenching, while the comparatively low number of precipitate-forming elements in alloy EN-AW-6016 makes it less sensitive to quenching variations. We also show that Sn reduces pre-aging kinetics. The optimal pre-aging temperature and time were consequently found to increase when Sn is added. We also studied the effect of adding a further thermal spike to the usual long-term pre-aging, at different positions within the processing route. The results we present are discussed based on a simulation of vacancy evolution in the alloy when subjected to these treatments.

## 1. Introduction

Al-Mg-Si alloys offer a variety of advantageous properties, such as good corrosion resistance, medium to high strength, and good formability [1]. Therefore, they are widely used in automotive applications, e.g. car body sheets [2,3]. Natural aging (NA) after solution aging and quenching, even for a duration of only a few minutes [4], causes a reduction in the strength achievable during subsequent artificial aging and a degradation of the hardening kinetics. This is caused by the formation of clusters, which cannot act as nuclei for the precipitation of the main hardening phase β’’ in Al-Mg-Si alloys. Though some of these NA-clusters are dissolved at the beginning of artificial aging (AA) (Si-rich clusters are thought to be not re-dissolved by AA [5]), solute content and vacancy concentration are reduced significantly [6]. On the one hand, pre-aging can serve to prevent hardening during NA. On the other hand, it promotes a greater increase in strength upon AA. For completeness, some minor alloyed 6xxx-materials can benefit significantly from natural aging [7,8].

Pre-aging (PA) parameters are a trade-off between time and temperature. The higher the temperature chosen, the shorter the applied dwell time has to be. Otherwise, the material will be strengthened too much, and the forming or stamping processes will be strongly inhibited in the pre-aged T4P state [9,10]. It was shown that 70 °C marks a boundary temperature. If clusters are formed above this, they can transform into β’’. Below, they cannot transform or act as a nuclei [11]. Observations from a 3D atom probe clearly reveal different types of clusters that form during either PA or NA. The crucial property of clusters, in terms of transformation in the course of AA, is claimed to be the Mg/Si ratio. While measurements show a balanced ratio of about one for PA-treatments, clusters tend to follow the overall composition of the alloy after NA [12,13,14,15,16]. Besides “conventional” pre-aging for several hours, applying a spike as a short-term treatment at high temperatures (180 °C and 200 °C) also shows promising results [6,17,18].

Studies on reversion heat treatments of naturally aged materials show the re-dissolution of formed clusters at the beginning of artificial aging. Applying a high temperature spike for a few seconds can restore a material state comparable to that after solution aging. This results in similar natural aging behavior afterwards [19]. It can also generate enhanced paint-bake performance [20].

Besides this already rather well-understood thermal treatment, an alloy variation including Sn offers a promising alternative to suppress undesired natural aging in Al-Mg-Si alloys [21]. This mechanism is based on high vacancy-solute binding energy, which hinders vacancies from travelling within the crystal and, thus, significantly impedes the movement of Mg and Si at room temperature (RT) [22]. During AA, at elevated temperatures, the bonds between Sn and vacancies are broken and Mg and Si can diffuse freely. This results in a comparatively great increase in strength without undesired natural aging effects [23]. It was also shown that Sn-alloying causes very fast artificial aging when aged right after solution aging [24]. Because Sn is active only in solid solution, solubility is a key factor. Studies have shown that both the three major alloying elements used for age hardenability (Mg, Si, and Cu) and the solution aging temperature influence the Sn solubility tremendously [25]. Whereas increasing the amount of Si to a large extent minimizes the solubility of Sn, Mg has only a minor influence. Regarding solution aging, it was found that the selected temperature should be as high as possible for a maximum amount of Sn in solid solution [25].

This study aimed to evaluate the influence of thermal treatment, in particular solution aging and pre-aging, on Sn-alloyed Al-Mg-Si alloys. Both hardness testing and electron microscopy as well as simulations were deployed to this end. To determine the impact of pre-aging parameters, extensive variation of temperature and time oriented toward established values was applied and the effect quantified via hardness testing.

## 2. Materials and Methods

Table 1 lists the alloys used for the different heating treatments in this study. They were produced industrially via hot and cold rolling by AMAG rolling GmbH, Ranshofen, Austria, and had a thickness of roughly 1 mm.

**Figure 1:**
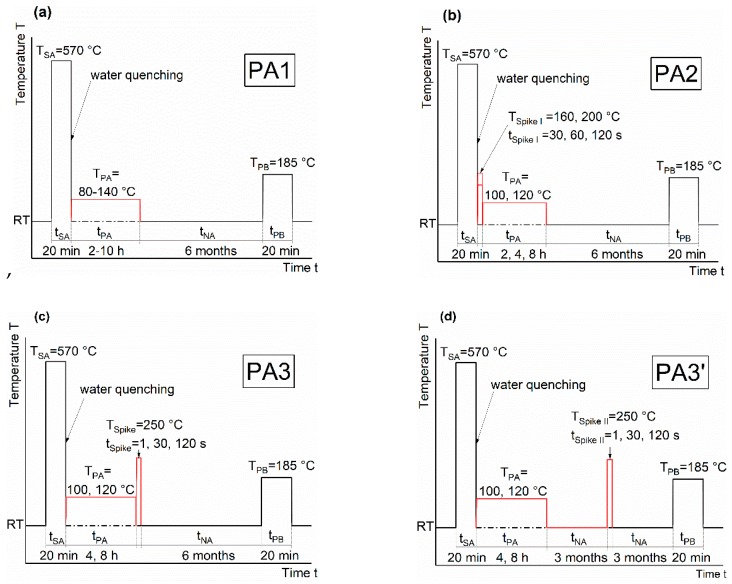
Overview of the applied pre-aging treatments PA1 (**a**), PA2 (**b**), PA3 (**c**), and PA3’ (**d**). Note that for each treatment, a reference was treated without pre-aging “As-Quenched” (dot-dashed black line).

Figure 1 schematically depicts the different pre-aging strategies that were applied. A detailed overview can be found in Table 2. All heat treatments started with solution annealing in a circulating air furnace (Nabertherm N60/85 SHA, Nabertherm, Lilienthal, Deutschland, Germany) at 570 °C, or 560 °C for HT0, lasting for 20 minutes. Quenching was conducted by immediately transferring the samples to a water bath. For comparison, air quenching was also performed within HT0. Pre-annealing (PA, 80 to 140 °C, 2 to 10 h), Spike I (160 to 200 °C, 30 to 120 s) and paint-bake simulations without prior deformation (185 °C, 20 min) were performed using an oil bath (Lauda Proline P 26, Lauda, Lauda-Königshofen, Germany). Due to the short dwell times and high temperature, Spike II was conducted in a metal bath consisting of Bi57Sn43. Natural aging samples were kept in a Peltier-cooled incubator IPP (Memmert, Schwabach, Germany) at 25 °C. The behavior of the samples during room temperature storage was tested over a period of six months via hardness testing (except for PA3’-samples, which were only tested for three months). After this, a paint-bake simulation (185 °C, 20 min) was applied and the final hardness was analyzed (note that, for better readability, not all hardness values are depicted in the graphs shown below). For Brinell hardness testing (HBW 2.5/62.5), an EMCO-Test M4 device (emcotest, Kuchl, Austria) was used. A maximum standard deviation of 2.0 HBW 2.5/62.5 was undershot.

To characterize the AlFeMnCrSi-phases and the precipitation of Sn-phases upon cooling, TEM measurements were performed using a FEI Talos F200X (ThermoFisher, Hillsboro, OR, USA). The latter was equipped with a Super-X EDS system while 200 kV was used as the acceleration voltage. Specimens were prepared via a standard route consisting of grinding and electrolytic-polishing with HNO_3_ in Methanol (1:3) at −30 °C and 12 V.

**Figure 2:**
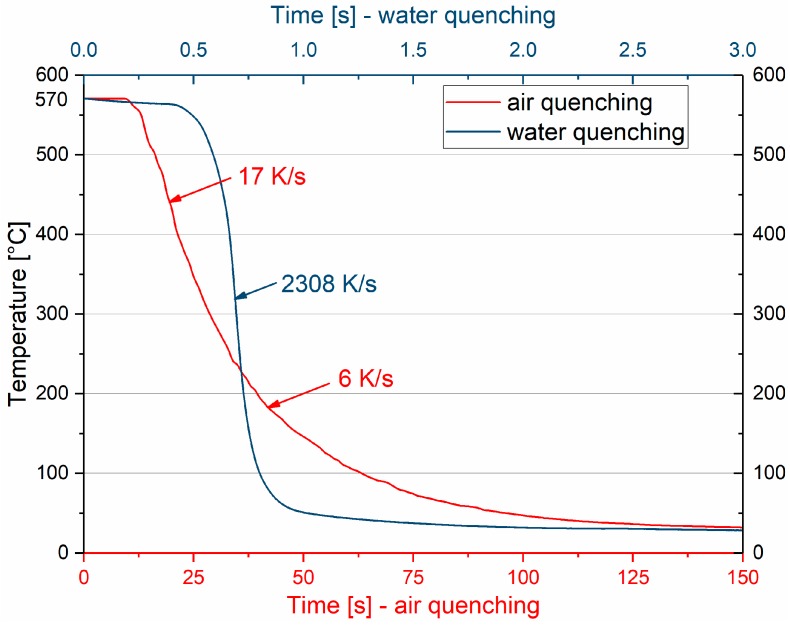
Cooling rate from 570 °C of 1-mm thick aluminum sheets with air or water quenching.

Thermo-kinetic simulations were conducted with MatCalc 6.00 (MatCalc Engineering, Vienna, Austria) [26]. First, equilibrium calculations were applied to determine the differing amounts of dispersoids within EN-AW-6016 and EN-AW-6061 at 570 °C. Second, kinetic simulations were deployed to understand the vacancy formation and annihilation during the various heat treatments using the FSAK (Fischer-Appel-Svoboda-Kozeschnik) vacancy annihilation model [27,28]. Trapping of vacancies on Sn was set active, using a binding energy of 0.24 eV, according to Reference [21]. A grain size of 30 μm and an equilibrium density of 10^11^ m^−2^ were assumed. Concerning heating, 220 K/s was used for heating within the metal bath ([19]) and 10 K/s as the heating rate inside the oil bath. For cooling, water quenching with 2000 K/s (see Figure 2) was assumed.

## 3. Results

### 3.1. Quench Sensitivity

Figure 2 shows the achievable cooling rates for the sheet samples used while quenching in water or air. Whereas quenching in water cools the sample to below a critical temperature of 300 °C in far less than a second (preventing pre-precipitations of Mg and Si), this takes about 20 s with air cooling.

**Figure 3:**
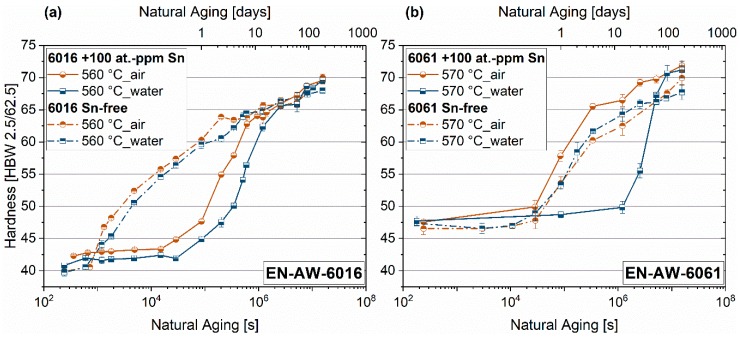
Natural aging behavior over six months of EN-AW-6016 and EN-AW-6061, which contains 100 at.-ppm Sn (solid line) and Sn-free (dot-dashed line). EN-AW-6016 (**a**) and EN-AW-6061 (**b**).

Figure 3a,b depict the influence of these two different quenching techniques on the hardness development of the 6xxx-alloys tested, with and without Sn. Figure 3a shows the natural aging behavior of EN-AW-6016. Adding Sn to this alloy type significantly delays the start of hardness increase during room temperature storage from 10 minutes to roughly 4 to 8 h. The onset of natural aging of the Sn-alloyed EN-AW-6016 is only slightly influenced by the two different quenching techniques. Figure 3b shows that 100 at.-ppm Sn-addition to EN-AW-6061 can generate a retardation of the natural aging start from 8 h to 14 days. However, such a delay can only be seen if the Sn-containing alloy is quenched with water. Air-quenching shows no altered natural aging behavior. Concerning the Sn-free samples, neither alloy type is sensitive to the different quenching technique with respect to their aging characteristics.

### 3.2. Pre-Aging 1

#### 3.2.1. Natural Aging

**Figure 4:**
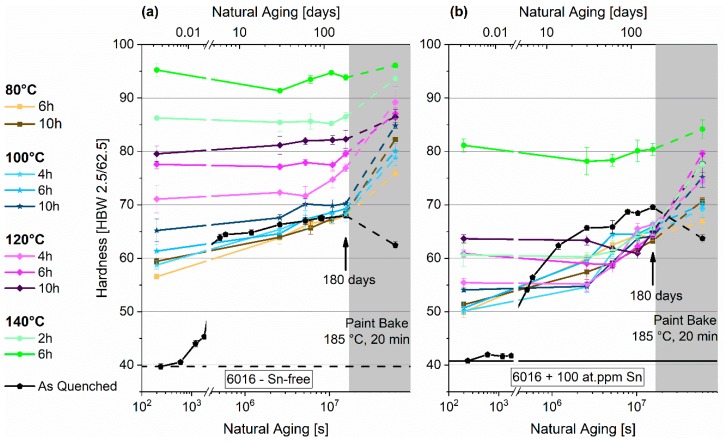
Natural aging behavior of samples treated according to PA1 for EN-AW-6016 Sn-free (**a**) and EN-AW-6016 + 100 at.-ppm Sn (**b**). The shaded areas show the hardness evolution during paint-bake treatment (185 °C, 20 min, no pre-deformation).

**Figure 5:**
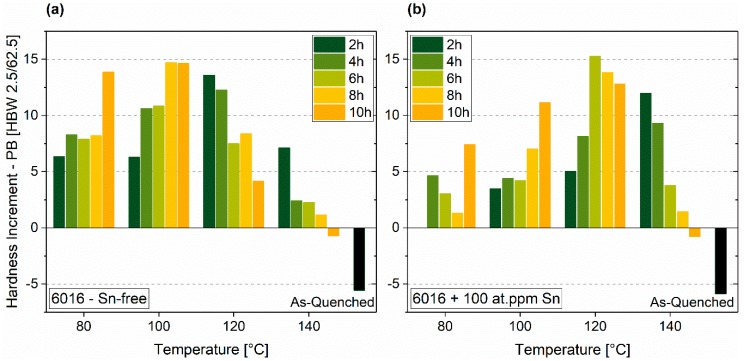
Paint-Bake response (185 °C, 20 min, no pre-deformation) of samples treated according to PA1 for EN-AW-6016 Sn-free (**a**) and EN-AW-6016 + 100 at.-ppm Sn (**b**).

Figure 4a,b show the differing hardening response of the two EN-AW-6016 alloys tested (with and without Sn) for PA at 80 °C to 140 °C for various durations. Generally, identical parameters generate a higher hardness response for the Sn-free specimens, shown in Figure 4a. To reach the same initial hardness after PA, the annealing temperature of the Sn-containing alloy must be increased by 20 °C. While the Sn-free alloy shows only a moderate hardness rise during NA for PA-temperatures below 120 °C, the Sn-containing alloy in Figure 4b exhibits distinct natural aging, starting from lower hardness values. Although nearly all samples show increasing hardness values, the start of natural aging is seen after one or two months for the Sn-containing alloy. After 180 days, all Sn-alloyed specimens show a comparable hardness of about 65 HBW. Concerning the Sn-free alloy, the different pre-aging strategies result in different hardness values, which are still present after 180 days of room temperature storage. Paint-bake simulation (185 °C, 20 min, no pre-deformation) produces a hardness drop in the as-quenched samples independent of Sn-addition (Figure 4 and Figure 5).

#### 3.2.2. Paint-Bake Simulation

Similar to natural aging, Sn-addition significantly influences the hardness increase on paint-bake. The Sn-free alloy shows a maximum increment of 14.7 HBW at 100 °C and 8 h of pre-aging (Figure 5a). For the Sn-containing alloy, the temperature must be increased to 120 °C to obtain the maximum rise in hardness of 15.3 HBW (Figure 5b). There is no significant difference in the maximum reachable paint-bake performance. Paint-bake results may suggest that an Sn-free EN-AW-6016 alloy is not as sensitive to PA as an Sn-added alloy. In other words, more time and temperature combinations generate hardness increments of 10 HBW or more for the Sn-free alloy.

### 3.3. Pre-Aging 2

#### 3.3.1. Natural Aging

**Figure 6:**
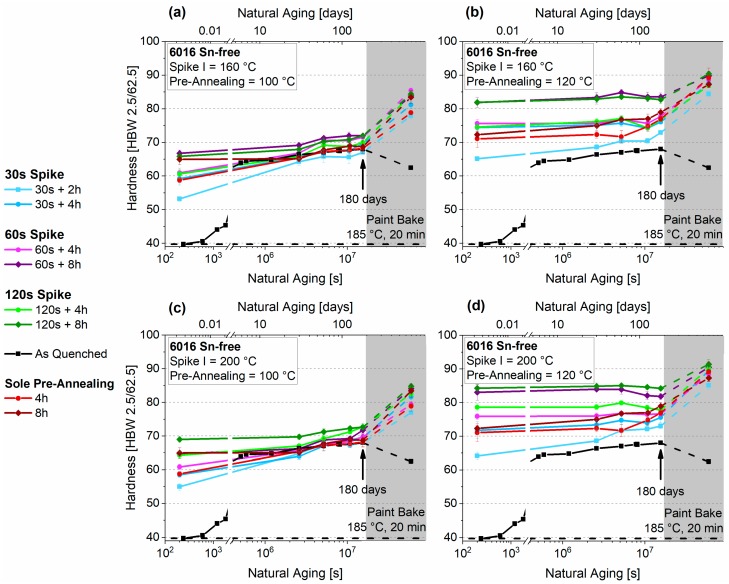
Natural aging behavior of samples treated according to PA2 for EN-AW-6016 Sn-free. Spike I 160 °C + PA 100 °C, (**a**) Spike I 160 °C + PA 120 °C, (**b**) Spike I 200 °C + PA 100 °C, (**c**) Spike I 200 °C + PA 120 °C (**d**). The values from PA1 without Spike I are also depicted in red. The shaded areas show the hardness evolution during the paint-bake treatment (185 °C, 20 min, no pre-deformation).

Figure 6a–d show the natural aging behavior of EN-AW-6016 without Sn, which undergoes PA2 consisting of Spike I (160 °C, 200 °C) + pre-annealing at 100 °C and 120 °C. The values of PA1 are also depicted in red for 4 h and 8 h of sole pre-annealing. Depending on the PA temperature, initial hardness differs significantly. Independent of duration, 100 °C pre-annealing produces hardness values below 70 HBW in the beginning, while still showing distinct natural aging. The hardness increase during RT (room temperature) storage is suppressed considerably after PA for eight hours. Samples treated at 120 °C show stable behavior, but starting from higher values. Concerning the spike treatments, a different outcome can be seen at 160 °C and 200 °C. While samples treated at 200 °C show increasing hardness values after longer spike durations (Figure 6c,d), there is no significant difference for treatment at 160 °C (Figure 6a,b). In addition, all initial hardness values are higher than those of the samples treated without Spike I in PA1.

**Figure 7:**
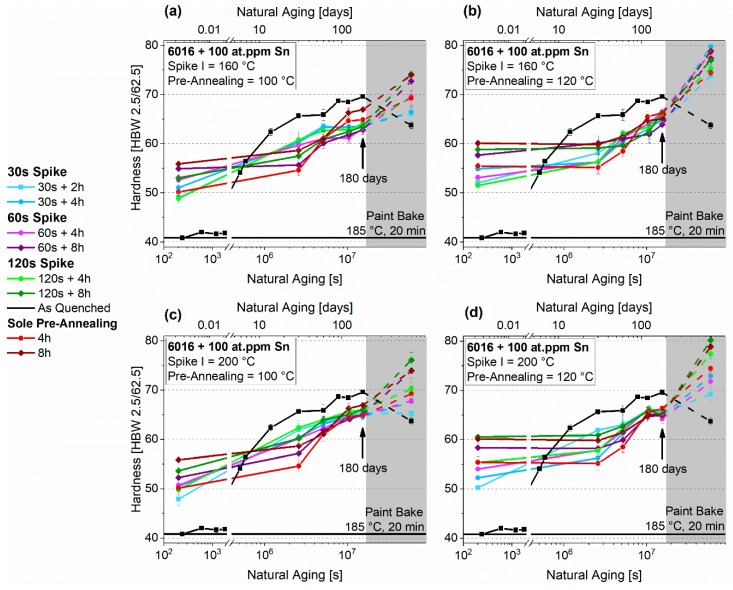
Natural aging behavior of samples treated according to PA2 for EN-AW-6016 + 100 at.-ppm Sn. Spike I 160 °C + PA 100 °C (**a**), Spike I 160°C + PA 120 °C (**b**), Spike I 200 °C + PA 100 °C (**c**), Spike I 200 °C + PA 120 °C (**d**). The values from PA1 without Spike I are also depicted in red. The shaded areas show the hardness evolution during the paint-bake treatment (185 °C, 20 min, no pre-deformation).

Figure 7a–d depict the natural aging behavior of EN-AW-6016 + 100 at.-ppm Sn, which is aged by a combined treatment consisting of Spike I (160 °C, 200 °C) + PA at 100 °C and 120 °C (PA2). In the context of initial hardness, the spike treatment has a minor influence compared to sole PA. Pre-annealing at 120 °C leads to a wider variation in hardness. After 180 days, these differences, which is prominent at the start, level out due to natural aging of nearly all samples. All initial hardness values are below those of the samples treated without Spike I in PA1. Although initial hardness is higher for samples treated according to PA1, they show more pronounced NA behavior, like samples from PA2, which also received a spike.

#### 3.3.2. Paint-Bake Simulation

**Figure 8:**
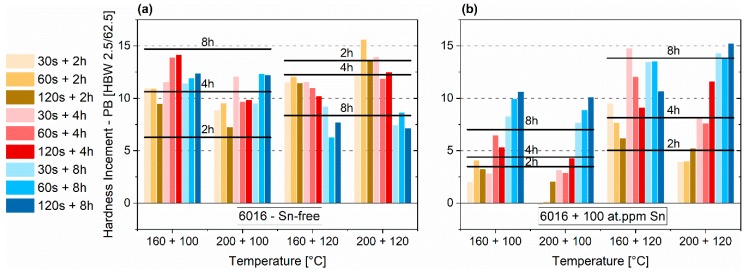
Paint-bake response (185 °C, 20 min, no pre-deformation) of samples treated according to PA2 (Spike I - first temperature in the axis label + PA - second temperature in the axis label) for EN-AW-6016 Sn-free (**a**) and EN-AW-6016 + 100 at.-ppm Sn (**b**). The horizontal black lines mark the paint-bake response of sole PA at 100 °C (left two column group) and at 120 °C (right two column group).

A spike treatment before pre-annealing can enhance the paint-bake performance in various cases (Figure 8a,b). The horizontal black lines mark the PB (paint bake) responses of samples that are pre-annealed solely at 100 °C or 120 °C. Concerning the EN-AW-6016 alloy without Sn, the spike seems to depict additional thermal energy that enhances the hardness increase during PB. This is only valid for pre-annealing at 100 °C. If pre-annealing is performed at 120 °C, a spike beforehand generates minor paint-bake performance (Figure 8a). Particularly, the combination of 160 + 120 °C shows increased increments compared to PA1 for the Sn-containing alloy (Figure 8b).

**Figure 9:**
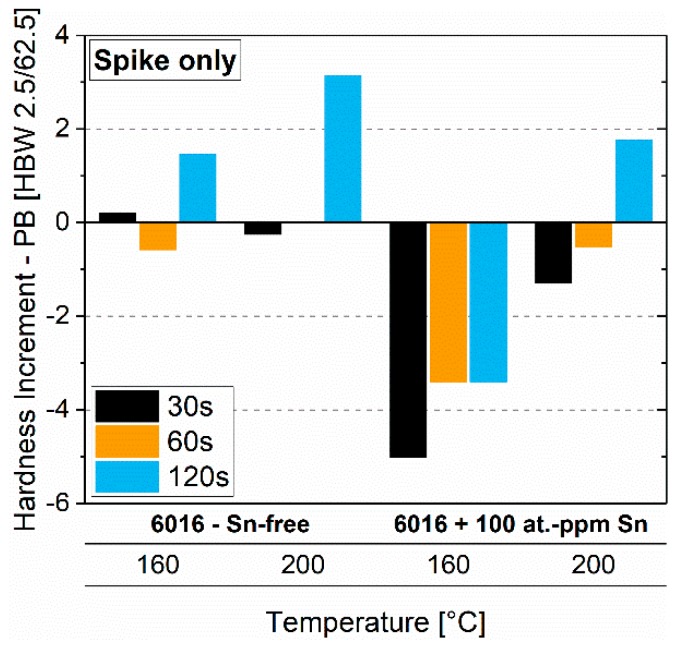
Paint-bake response (185 °C, 20 min, no pre-deformation) of samples treated solely with Spike I at 160 °C and 200 °C (EN-AW-6016 Sn-free and EN-AW-6016 + 100 at.-ppm Sn).

Figure 9 shows the paint-bake response (185 °C, 20 min, no pre-deformation) of samples of both alloys treated by Spike I (160 °C, 200 °C) only. While this treatment generates a hardness increase for the Sn-free alloy after 120 s independent of temperature, a hardness increase only occurs for 120 s at 200 °C for the Sn-containing alloy. Moreover, it is seen that longer treatment at a higher temperature increases the paint-bake response and, in the end, turns the values from negative to positive (Figure 9, Sn-containing).

### 3.4. Pre-Aging 3

#### 3.4.1. Natural Aging

**Figure 10:**
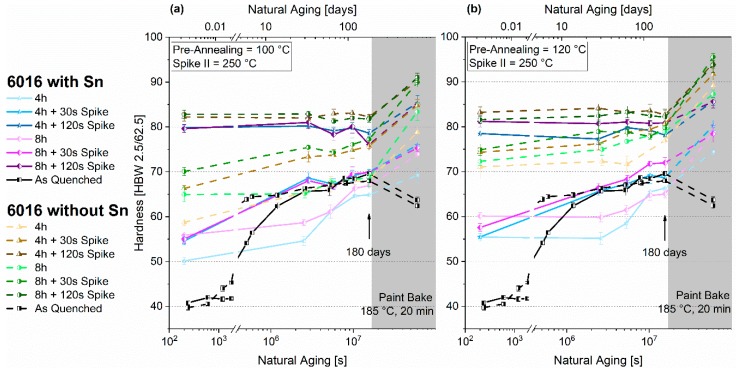
Natural aging behavior and paint-bake response of samples treated according to PA3 for EN-AW-6016 Sn-free and EN-AW-6016 + 100 at.-ppm Sn PA at 100 °C + Spike II 250 °C (**a**) and PA at 120 °C + Spike II 250 °C (**b**). The shaded areas show the hardness evolution during the paint-bake treatment (185 °C, 20 min, no pre-deformation).

Figure 10a,b depict the hardness evolution during room temperature aging of EN-AW-6016 with and without Sn after applying PA3. It is revealed that Sn restricts the hardness increase upon pre-annealing, which is similar to other treatments. However, a spike at 250 °C shifts the hardness to higher values, depending on the dwell time. A spike time of 30 s accelerates natural aging of the Sn-containing specimens significantly. Even after six months, a distinct discrepancy is still prominent.

**Figure 11:**
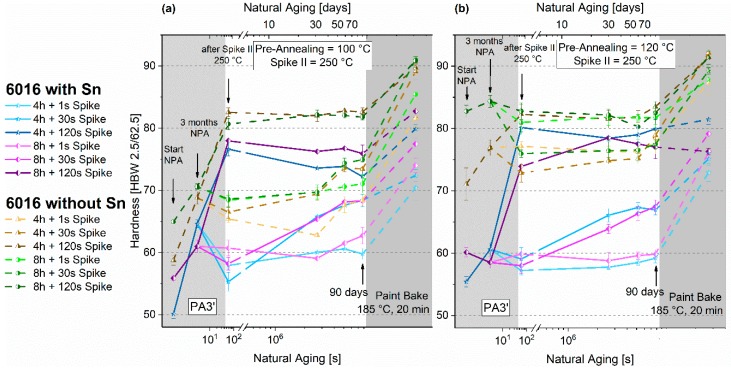
Natural aging behavior and paint-bake response of samples treated according to PA3’ for EN-AW-6016 Sn-free and EN-AW-6016 + 100 at.-ppm Sn. PA at 100 °C + Spike II 250 °C (**a**) and PA at 120 °C + Spike II 250 °C (**b**). The shaded areas depict the hardness behavior before application of Spike II. The shaded areas show the hardness evolution during the paint-bake treatment (185 °C, 20 min, no pre-deformation).

Figure 11a,b depict the results of PA3’, which evaluates the influence of three months of natural pre-aging (NPA) after pre-annealing before applying Spike II to the hardness. Depending on the initial PA time and temperature, the values follow the same characteristics as already shown in Figure 4 for PA1. Applying Spike II shows different outcomes. 1-second and 30-s dwell time of the spike result in a hardness decrease or at least cause stagnation. 120 s lead to a drastic increase in hardness, except for the Sn-free alloy after eight hours of pre-annealing at 120 °C. During room temperature storage, nearly all samples show a stable condition. A drastic re-increase of the hardness value is observed after a 30-s spike.

#### 3.4.2. Paint-Bake Simulation

**Figure 12:**
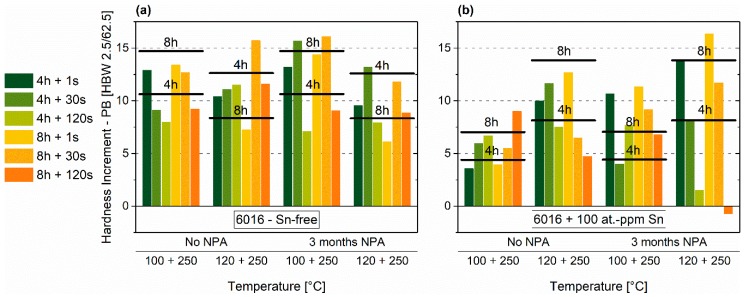
Paint-bake response (185 °C, 20 min, no pre-deformation) of samples treated according to PA3 (no NPA) and PA3’ (3 months of NPA before applying Spike II) (pre-annealing - first temperature in the axis label + Spike II - second temperature in the axis label) for EN-AW-6016 Sn-free (**a**) and EN-AW-6016 + 100 at.-ppm Sn (**b**). The horizontal black lines mark the paint-bake response of sole pre-annealing at 100 °C and at 120 °C.

Figure 12a,b depict the results of the paint-bake simulation at 185 °C for PA3 and PA3’. The horizontal black lines mark the PB responses of samples that are pre-annealed solely at 100 °C or 120 °C. Similar to the other PA strategies, the Sn-modified alloy is more sensitive to the various combinations of temperature and time of spike as well as pre-annealing. However, the maximum increment of roughly 16 HBW is reached with both alloys. 

In the Sn-free alloy, the maximum hardness increments are realized upon 30-s spike treatments. The best results are observed for 100 °C pre-annealing. The combination of 8 h PA at 120 °C and a 30 s Spike II (PA3’) shows a maximum increase. For comparison, PA1 at 120 °C for 8 h proved to be too high a temperature (see Figure 5).

The Sn-containing samples show similar values when compared to the only pre-annealed material (see Figure 5). Especially with 120 °C pre-annealing, over-aging effects occur when long Spike II treatments are applied.

## 4. Discussion

### 4.1. Quench Sensitivity

Sn alloying only leads to a retardation of natural aging when it is present in solid solution. It was already shown that the main hardening elements of 6xxx-alloys have a detrimental influence, since Mg and Si lower the maximum quenchable Sn amount in solid solution [23].

Figure 3a shows the natural aging behavior of EN-AW-6016 with and without Sn. In general, the different quenching techniques applied have no influence on the onset of hardening in these alloys, and Sn addition retards hardening. In EN-AW-6061, the effect of 100 at.-ppm of Sn vanishes when air cooling is applied (Figure 3b). Note that the higher Si-content in EN-AW-6016 may lead to a significant acceleration of natural aging in general [16,23,29].

**Figure 13:**
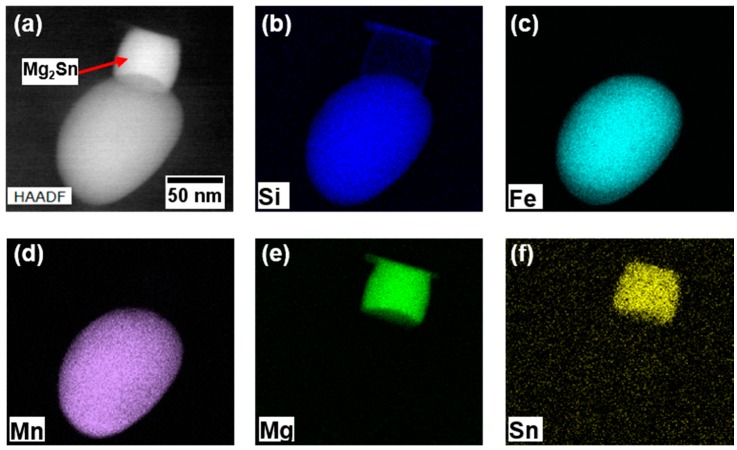
STEM HAADF image of EN-AW-6016 + 100 at.-ppm Sn (**a**) and corresponding EDX mappings of Si (**b**), Fe (**c**), Mn (**d**), Mg (**e**), and Sn (**f**). Mg_2_Sn is precipitated upon air quenching after solution annealing (560 °C) at the incoherent interface of an Al(Fe,Mn)Si-phase.

We suggest that the different quench sensitivity of these two alloys is linked to the intermetallic phases (IMP)-forming elements Fe, Mn, and Cr. Figure 13a shows a STEM-HAADF image of an Fe, Mn, Si-rich IMP, present in EN-AW-6016 + 100 at.-ppm Sn after air quenching. Figure 13b–f show the corresponding EDX mappings of Si, Fe, Mn, Mg, and Sn. The round particle at the bottom can be identified as an Al(Fe,Mn)Si-phase. Typically, these precipitates have an incoherent interface with the aluminum matrix [30,31]. The rectangular particle, sitting on top of the roundish Fe, Mn, Si-phase, is identified as Mg_2_Sn (Figure 13e,f). It is well known that IMPs can serve as nucleation sites for Mg,Si-hardening phases [32]. Here, we show that Mg, Sn-phases also nucleate at IMPs during quenching. Thus, the amount of Sn in solid solution is reduced, which lowers the natural aging retardation to a certain extent.

**Figure 14:**
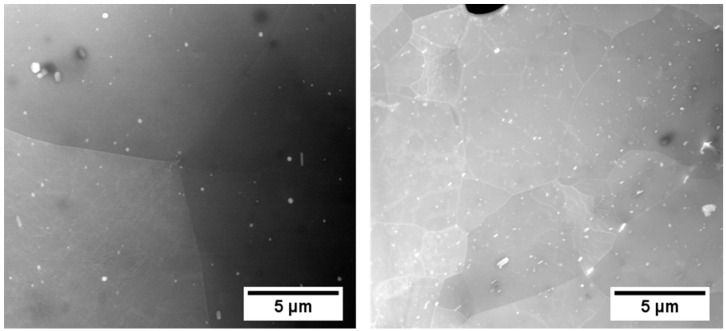
STEM HAADF images of EN-AW-6016 + 100 at.-ppm Sn (**a**) and EN-AW-6061 + 100at.-ppm Sn (**b**). Both samples were solution annealed at 560 °C (6016) or 570 °C (6061) and quenched via air cooling. The number of visible IMPs is much higher for EN-AW-6061 (**b**), due to higher Fe, Mn, and Cr content.

Figure 14 shows the microstructure of EN-AW-6016 (a) and EN-AW-6061 (b), which both contain 100 at.-ppm Sn. It is evident that the number of bright intermetallic particles is much higher for EN-AW-6061 due to a higher content of IMP-forming elements such as Fe, Mn, or Cr (see Table 1). Equilibrium calculations with MatCalc at 570 °C show different amounts of the AlFeMnCrSi-phase. EN-AW-6016 contains 0.61 wt.-% of this phase, whereas 1.5 wt.-% of AlFeMnCrSi is present in EN-AW-6061. Therefore, EN-AW-6061 + 100 at.-ppm Sn is much more sensitive to the quenching rate. This results in a different onset of hardening upon natural aging (Figure 3b), as it contains more potential nucleation sites for the precipitation of Sn-containing phases. Only after water quenching can vacancy trapping by sufficient Sn in solid solution occur. On the other hand, Sn-containing EN-AW-6016 shows no different aging characteristics at room temperature (Figure 3a) because the number of incoherent intermetallic phases is quite low and the quenching procedure is less critical.

### 4.2. Conventional Pre-Aging (PA1)

Hardness upon pre-annealing and during subsequent room temperature storage reveals significant differences between Sn-free and Sn-containing EN-AW-6016 (Figure 4). Since the prevalent hardness increase is attributed to cluster formation, excess vacancy concentration and its mobility are key factors. Considering the high vacancy binding energy of Sn, a lower hardness after pre-annealing of the Sn-containing samples seems reasonable. Sn retards cluster formation during pre-annealing due to a mobility decrease of vacancies, as observed in Reference [33]. To reach the same initial hardness after pre-aging, more heat energy must be applied to the Sn-containing samples. This is reasonable because additional thermal activation needs to be overcome to release vacancies trapped at Sn atoms [29]. Depending on the initial hardness after PA1, natural aging is observed for both alloys (Figure 4a,b). Although Sn retards cluster formation during pre-annealing, clusters continue to form at RT, which means that Sn cannot fully suppress the vacancy-aided cluster-formation process over a period of six months of RT-storage after pre-annealing. Whereas, for Sn-free samples, NA is observed right from the start (Figure 4a). Sn-containing samples show a constant hardness level before distinct NA occurs (Figure 4b). We assume that a certain number of Sn-vacancies pairs survive the PA treatment. Lower hardness after PA means a lower degree of cluster formation and, thus, a higher number of Sn-vacancies pairs at RT. The higher their number, the more natural aging occurs due to a higher excess-vacancy content. At high PA temperatures and long PA times (i.e., 140 °C/4 h), however, clustering is well advanced. Consequently, the quenched-in vacancies may be exhausted to the level of equilibrium vacancy concentration and only a few non-clustered solutes remain. This situation is assumed to result in an absence of NA.

Considering the paint-bake performance of the alloy with Sn (Figure 5a) and without Sn (Figure 5b), similar maximum hardness increments were reached. However, the optimum time-temperature combination is more limited for the Sn-containing alloy. This alloy requires a higher PA temperature, which is attributed to the vacancy diffusion-limiting effect of Sn and reduced cluster formation. Both alloys exhibit a comparable maximum paint-bake response, but at different PA temperatures. We assume that the condition concerning cluster properties of the Sn-containing alloy with PA at 120 °C is comparable to the Sn-free case at 100 °C pre-annealing. “Beneficial clusters” ([14]) would determine the paint-bake response, independent of Sn-addition.

The observed decrease in hardness of the as-quenched and natural aged sample upon the paint-bake is caused by dissolution of clusters. This oft-reported behavior occurs at the beginning of artificial aging and is known as reversion [5,19,20]. Additionally, in our case, the paint-bake process can be seen as a shortened artificial aging treatment. An optimized bake hardening response requires a particularly suitable PA treatment.

### 4.3. Thermal Spike Plus Pre-Annealing (PA2)

The duration of the pre-annealing treatment mainly affects hardness. The Sn-free samples show some dependence on Spike I in their hardness after the entire PA2 treatment. Whereas the values do not differ after a spike at 160 °C, independent of the spike duration (Figure 6a,b), the hardness values increase upon longer spike treatments at 200 °C ((Figure 6c,d). The situation is reversed for the Sn-containing EN-AW-6016 (Figure 7), where, in most cases, a spike leads to a reduction of the initial hardness compared to PA1. However, starting from lower hardness, a more pronounced natural aging is observed during the first month.

We now consider two important aspects, which may be important for clustering behavior during PA2: (i) the “thermal load” introduced by the pre-treatments and (ii) the vacancy concentration during PA2.

Table 3 gives an overview of calculated Larson-Miller parameters for the PA and spike treatments used. Such calculations, which are based on the Arrhenius relation [34], give a rough comparison of different temperature-time combinations taking equilibrium diffusion into account only. The result suggests that the short spikes applied represent a thermal load for thermally activated processes on the material similar to that of the PA treatment. However, it must also be considered that the spike temperature is in the same range as the solvus temperatures of Si,Mg-co-clusters, which, for 6016, were calculated to be roughly 130 °C [35] or higher at 190 °C [28]. However, these are only rough estimations. One might assume that, at high spike temperatures, the driving force is weak or nonexistent for reactions taking place during low temperature pre-annealing.

**Figure 15:**
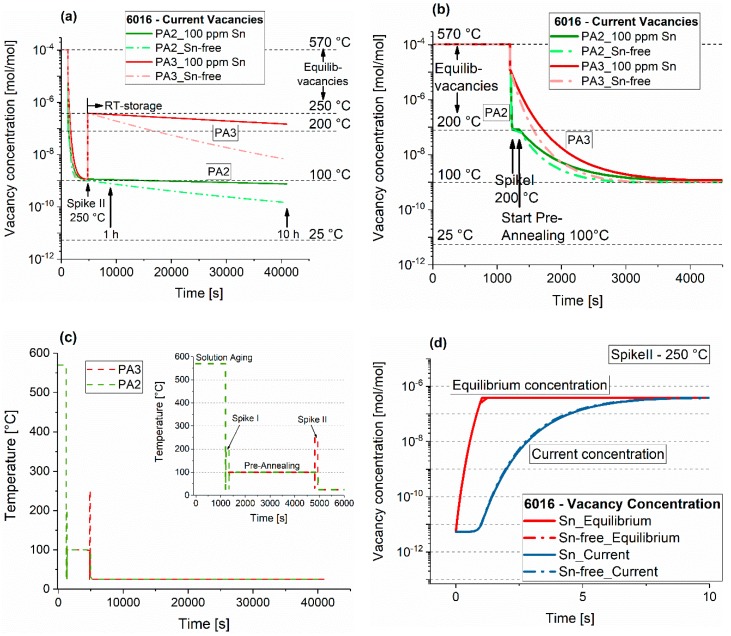
(**a**) Vacancy concentration during PA2 and PA3. The dashed horizontal lines represent the equilibrium concentrations at the respective temperatures. For better visibility, only one hour of pre-annealing at 100 °C was applied and 120 s for both Spike I (200 °C) and Spike II (250 °C). (**b**) An excerpt of (**a**) for better visibility. (**PA2:** Solution Aging → Quenching → Spike I (200 °C, 120 s) → Pre-Annealing (100 °C, 1 h) → RT. **PA3:** Solution Aging → Quenching → Pre-Annealing (100 °C, 1 h) → Spike II (250 °C, 120 s) → RT) (**c**) Detailed depiction of the heat treatment PA2 and PA3, which was used for vacancy calculations. (**d**) Vacancy generation upon heating from 25 to 250 °C with and without the trapping effect of Sn (heating rate 220 K/s). The dot-dashed lines mark the values of the Sn-free alloy. Calculated with MatCalc 6.00, using the FSAK vacancy annihilation model [27].

Figure 15a shows the calculated concentration of vacancies during PA2 (calculated for Spike I at 200 °C). After solution treatment, the vacancy concentration is kept at a high level due to water quenching. However, the application of Spike I at 200 °C leads to faster reduction in the vacancy density compared to sole application of PA at 100 °C (Figure 15b). The results do not indicate that this difference in vacancy concentration affects clustering, i.e., in the Sn-free case, the hardness after pre-annealing increases with application of a spike, which is counter-intuitive. During pre-annealing, it only takes about half an hour before the equilibrium vacancy concentration is reached. During storage at RT, the effect of Sn-trapping is prominent, which generates different vacancy annihilation speeds. For all calculations, cluster formation causing trapping of vacancies is not considered. This might play an important role, considering the significant disagreement between observed hardening kinetics and the calculated vacancy evolution upon pre-aging.

Concerning Spike I, the differences in hardness after pre-annealing cannot be explained by different vacancy concentrations, or, hence, by the affected clustering kinetics. The equilibrium vacancy concentration at 160 °C or 200 °C is already reached after 30 s. Consequently, from that point of view, each spike treatment is equivalent. Therefore, it can only be caused by cluster formation during the spike treatment, which contradicts the previously mentioned calculations of the solvus temperature of co-clusters. These considerations are only valid for the Sn-free alloy, as there is no hardness increase for the Sn-containing alloy. EN-AW-6016 + 100 at.-ppm Sn reacts to the spike by decreasing the initial hardness. In this case, the increased mobility of solute atoms during Spike I may promote the creation of Sn-aggregate-vacancy bonds, which can decrease clustering during subsequent pre-annealing.

Considering the calculations from Figure 15a, the vacancy concentration is kept at a higher level after PA2 for the Sn-containing alloy. Because Sn-vacancy bonds do not seem stable enough over six months, Sn-containing samples show distinct natural aging if they start from a low initial hardness value (Figure 7a–d)). For the Sn-free samples, natural aging occurs after low initial hardness and vanishes if pre-aging produces a high enough start hardness.

Paint-bake simulation of the Sn-free alloy shows the same tendencies as the values of PA1 (Figure 8a). Spike I at 160 °C generates an increase after four hours at 100 °C. The maximum value, which is reached after 100 °C pre-annealing in PA1, shifts to Spike I (200 °C) + 120 °C. For 120 °C of pre-annealing, decreasing hardness increments symbolize over-pre-aging, with Spike I. Considering the Sn-containing samples (Figure 8b), the hardness increase is comparatively small for samples pre-annealed at 100 °C. PA at 120 °C shows the best values. After four hours at 120 °C, Spike I at 160 °C increased the paint-bake response significantly, with the same initial hardness and similar natural aging extent as PA1.

Figure 9 shows that a spike alone at a sufficiently high temperature or a long enough time can turn a negative paint-bake response (as-quenched samples) into a hardness increase. The difference between Sn-free and Sn-containing is caused by different clustering kinetics during the spike treatment. Even though, according to the Larson-Miller parameters spike and PA depiction of a similar thermal load, our results show that clustering does not happen in a similar way during different treatments. Presumably, this is caused by temperatures above the cluster-solvus temperatures and reduced super-saturation.

### 4.4. Pre-Annealing Plus Subsequent Thermal Spike (PA3)

For discussion, the vacancy concentration during PA3 is also depicted (Figure 15a). It shows a similar trend to PA2, but without increased annihilation due to the absence of Spike I (Figure 15b). The application of Spike II (PA3, right after pre-annealing) generates a drastic re-increase in the vacancy content, which is only slowly annihilated at room temperature. This causes the observed distinct subsequent natural aging of the samples treated with a thermal spike of 30 s (see Figure 10a,b), while it does not appear in sole pre-annealed samples. This result applies to both alloys. However, it is more effective in the Sn-containing samples, presumably because of a significantly higher vacancy trapping of Sn after Spike II (Figure 15a). This higher content of surviving vacancies leads to a higher cluster formation over prolonged time at RT than in the Sn-free samples, as already seen in Figure 7c,d. 

Figure 15c shows the details of vacancy formation during heat treatment at 250 °C (Spike II). Independent of the Sn content, it takes about eight seconds before the equilibrium amount of vacancies is generated. The calculations suggest that a very short Spike II (i.e., 1 s) does not greatly influence the vacancy content, which is in line with the results in Reference [19]. Besides this, Banhart et al. [19] found considerable reversion of clusters for a short 1-second spike at 250 °C. The finding is as follows: no new vacancies are formed, but clusters are dissolved and the solute content is increased. For PA3’ (Figure 11a,b), a 1-second spike at 250 °C was applied after PA at 100 °C and 120 °C plus three months of NPA. A 1-second spike generates a drastic hardness decrease if the samples showed NPA. We assume that this reversion only refers to the hardness increase, which took place during NPA, i.e., only NPA-clusters are dissolved, but none that were formed by PA. However, the most important is the fact that no subsequent natural aging takes place after the 1-second spike reversion, which accords fully with the calculations. This is also comparable to results from interrupted aging, where NA is also suppressed [36]. Similar to PA3, a spike of 30 s initiates natural aging since this time is sufficiently long to generate new vacancies. 

Longer spike treatments of 120 s at 250 °C can already cause tremendous hardening upon PA3 and PA3’. This effect is comparable to the ultrafast aging observed in Reference [24]. Only for the Sn-free alloy pre-annealed at 120 °C for 8 h does the hardness drop slightly upon a 120-second spike treatment. Yet hardness is already at a high value after NPA.

The results from paint-bake simulation (Figure 12) show no new increases in hardness increments, which have been found upon a spike after natural aging [20]. Samples without Sn (Figure 12a) show no increased responses for PA3. Concerning PA3’ (including three months of NPA), the paint-bake response shows an increase or at least stagnation compared to PA1-values for a 1-second and 30-second spike. This is caused by the dissolution of non-transformable clusters by Spike II. This type of clusters forms during natural aging and causes the paint-bake response to deteriorate by decreasing the solute content [16,29].

Spike II application to the Sn-containing alloy can increase or decrease the hardness increment during paint-bake simulation, depending on pre-annealing (Figure 12b). Because 250 °C is above the solvus temperature for cluster formation, no additional clusters can be formed during the spike treatment. After a longer pre-annealing treatment, it is reasonable that more clusters are being dissolved, and, therefore, the paint-bake response decreases for this treatment. Regarding PA3’, including three months of NPA, the 1-second spike dissolves non-transformable clusters from NPA. In this way, the solute content is re-increased to higher values, which enables a better paint-bake response. A high hardness increment is also favored by the absence of natural aging after Spike II. A 30-second spike also causes dissolution, visible in the hardness decrease (Figure 11b). Yet, this is also accompanied by natural aging after Spike II. Therefore, no enhanced paint-bake performance is observed.

## 5. Conclusions

The aim of this study was to examine the behavior of Sn-containing and conventional Al-Mg-Si alloys upon different thermal treatments. Those consisted of quenching and various pre-aging strategies, including long-term pre-annealing and short-term spikes. Concerning pre-aging parameters, temperature-time combinations were chosen according to industrial values (PA1) and were modified by short-term spikes at different positions within the treatment, with respect to industrial applicability. Using hardness measurements, natural aging and paint-bake simulation characteristics were examined. Electron microscopy and computer simulations helped interpret the results. The following conclusion can be drawn.

- Sn-containing alloys are sensitive to the quenching technique where there are large numbers of incoherent particles in the material. If the quenching rate is too slow, Sn forms precipitates and is no longer active as a vacancy trap.
- Sn decreases clustering kinetics during pre-aging. Therefore, the temperature must be increased by at least 20 °C from 100 to 120 °C to obtain a hardness increase similar to that of Sn-free alloys. In this way, natural aging is suppressed and a comparable hardness increase during the paint-bake simulation can be realized.
- A high-temperature spike (250 °C) alters the natural aging behavior. Applied after three months of natural aging of pre-annealed material, it can lead to a reversion and will either accelerate or suppress subsequent natural aging.
- A spike of a few seconds can only dissolve clusters. More than 8 s are required to reach the equilibrium vacancy concentration at 250 °C, which accelerates subsequent natural aging.
- Among all tested heat treatment combinations, many of them showed promising hardness increases upon paint-bake simulation, which lie on a similar level. Yet, the question arises if other pre-aging treatments could lead to enhanced results. Notably, the Sn-containing alloy exhibited a good paint-bake response, even though the hardness after pre-aging stays at lower values compared to the Sn-free alloy.

## Figures and Tables

**Table 1:** Chemical composition of the EN-AW-6016 alloys used with and without 100 at.-ppm Sn and EN-AW-6061 with 100 at.-ppm Sn.

	[wt.-%]	Mg	Si	Cu	Sn	Fe	Mn	Cr	Zn	Ti	Al
6016	Sn-free	0.36	1.06	0.08	0.002	0.17	0.08	0.00	0.01	0.02	residue
	+100 at.-ppm Sn	0.40	1.08	0.07	0.042	0.18	0.06	0.01	0.01	0.02	residue
6061	Sn-free	0.83	0.48	0.37	0.002	0.46	0.11	0.16	0.05	0.01	residue
	+100 at.-ppm Sn	0.81	0.59	0.23	0.040	0.46	0.11	0.16	0.05	0.05	residue

**Table 2:** Detailed overview of each heat treatment.

	Solution Aging		Quenching Method	Spike I		Pre-Annealing		Spike II	
	T [°C]	t [min]		T [°C]	t [s]	T [°C]	t [h]	T [°C]	t [s]
**HT0**	560/570	20	air/water	-		-		-	
**PA1**	570	20	water	-		80/100/ 120/140	2/4/6/8/10	-	
**PA2**	570	20	water	160/200	30/60/ 120	100/120	2/4/8	-	
**PA3/PA3’**	570	20	water	-		100/120	4/8	250	1/30/ 120

**Table 3:** Comparison of Larson-Miller parameters of pre-annealing and spike parameters. Values were calculated according to Larson-Miller parameters = T [K] * (log(time [h]) + C). Constant C was set to 13.9 for all calculations, according to Reference [34].

Pre-Annealing				Spike			
		T [°C]				T [°C]	
		100	120			160	200
**t [h]**	**2**	5299	5583	**t [s]**	**30**	5120	5593
	**4**	5411	5701		**60**	5251	5735
	**8**	5524	5820		**120**	5381	5878
